# Commentary: Olfactory aversive conditioning during sleep reduces cigarette-smoking behavior

**DOI:** 10.3389/fpsyg.2015.00586

**Published:** 2015-05-06

**Authors:** Nicola Cellini, Valentina Parma

**Affiliations:** ^1^Department of General Psychology, University of PadovaPadova, Italy; ^2^Monell Chemical Senses CenterPhiladelphia, PA, USA

**Keywords:** aversive therapy, behavioral change, learning, odors, sleep

For more than half of a century, we have known that the sleeping brain is able to perceive sensory information; however, learning new associations during sleep was deemed mythical. Indeed, experiments suggested that participants could not recall material presented during EEG-monitored sleep (Simon and Emmons, 1956). Recently, Arzi et al. (2012) made an exciting discovery, which suggested a revision of the myth. They demonstrated that humans can indeed learn new associations while they are asleep and act on this knowledge both during sleep and the ensuing wake period. Arzi et al. (2012) paired non-awakening pleasant and unpleasant odors with sounds during sleep. When the association was acquired, they measured sniff responses to the sounds only. As expected, inhalations to the tones matched with pleasant odors were stronger than those paired with malodors, demonstrating that healthy young adults can acquire novel associations while sleeping and maintain them during wake. Recently, the same group built on this groundbreaking discovery by addressing a serious health concern, namely smoking cessation. Capitalizing again on the valence-dependence of the sniff response, they paired cigarette odor with aversive smells during sleep in a group of nicotine-addicts who subsequently significantly reduced the number of cigarettes smoked in the week following exposure (Arzi et al., 2014). In these studies, Arzi et al. (2012, 2014) took advantage of the distinctive, yet complementary, features of sleep and olfaction. Compelling evidence indicates that sleep may be optimized for the cortical reorganization that mediates memory consolidation (Rasch and Born, 2013), in part because of the reduced sensory input during this brain state. As sleep deepens, thalamo-cortical neurons show increased hyperpolarization that limits sensory inputs to the cortex (Steriade, 2003). However, odors might be exceptional stimuli for two reasons: first, the lack of a necessary thalamic relay offers odors privileged cortical access; and second, during slow wave sleep, functional connectivity between olfactory, limbic, and neocortical areas is enhanced (Barnes and Wilson, 2014). With this in mind, Arzi et al. (2014) transformed odors into Trojan horses, sneaking into the sleeping brain and creating associations between cigarettes and noxious smells. As a result, the idea of smoking during wake becomes, with no voluntary effort, as unsavory as it was during encoding while asleep. Although fears outside of the full awareness domain are known for being quickly acquired and swiftly forgotten (Lovibond and Shanks, 2002), the behavioral changes resulting from this sleep learning study lasted at least a week. Importantly, this surprising, long-term, positive health outcome was achieved with only one odor-induced sleep learning session and without mustering willpower or emotional resources.

Tapping into non-voluntary learning processes for clinical purposes gains further plausibility considering the independent findings by Hauner et al. (2013). In this case, the authors presented during wake a visual aversive conditioning in the presence of an olfactory context. Odors previously matched with a threatening stimulus, such as an irritating electrical shock, and then presented alone during slow wave sleep, facilitate human psychophysiological and neurophysiological processes underpinning fear extinction. Very recently, this form of inhibitory learning has also been extended to auditory triggers (He et al., 2015).

Thus, re-presenting learned odors while asleep is enough to recall related emotional material and may unconsciously modify the old association with the new (non-traumatic) experience during sleep. In humans, the beneficial effects of acquiring (Arzi et al., 2014) or just re-targeting emotional memories during sleep (Hauner et al., 2013; He et al., 2015) could address, in an evidence-based manner, disorders such as alcoholism, multiple chemical sensitivity, specific phobias, washing and cleaning compulsions, trauma-related, and eating disorders, for which many treatments entail highly distressing side effects. For instance, considering that conditioned aversion therapy for alcoholism has shown mixed results (O'tousa and Grahame, 2014), we hypothesize that the same treatment during sleep might produce more convincing outcomes toward the maintenance of sobriety. Along the same lines, intense fear reactions, such as those experienced by patients with phobias or post-traumatic stress disorder (PTSD), often prevent clinicians from using exposure therapy tools or significantly reduce compliance and/or increase the time at which clinical improvement is achieved. Exposing patients during sleep would maintain the “exposure” component of the treatment—critical in securing improvement—and it would reduce collateral overt emotional burden, for both patient and therapist.

Such tempting speculations underscore the need for more robust empirical evidence and demand that many important basic questions regarding mechanisms are addressed before clinical implementation. For example, potential unpredicted side effects of such a treatment are not known. Indeed, the re-targeting process of fear memories provide opposite outcomes across species. In humans, cueing memories during SWS (Hauner et al., 2013; He et al., 2015) extinguish fear responses, whereas in rodents fear memories are enhanced (Rolls et al., 2013; Barnes and Wilson, 2014). As argued by Diekelmann and Born (2015), although it is more likely that these differential effects depend on the methodological differences of this set of studies rather than on species-specific mechanisms, further research is warranted in order to clarify this issue.

It is also unknown whether aversive conditioning during sleep specifically modifies the hypothesized behavior(s) alone (e.g., smoking) or some downstream effects on ancillary undesired outcomes (e.g., increased food cravings). Furthermore, would the minimal awareness of olfactory experiences and the lack of clear olfactory counterparts for many mental representations (Stevenson, 2009) represent stumbling blocks for the translation beyond the laboratory? Also, the temporal dimension of the outcomes needs to be defined. Evidence from aversive olfactory learning during wake suggests that perceptual salience effects following one-time exposure are present a week following conditioning, but disappear 8 weeks later, therefore suggesting that this type of learning has specific time constraints (Åhs et al., 2013). As a consequence, longitudinal studies following up volunteers for more than a week post-sleep learning (ideally 6–12 months for clinical purposes) are warranted to assess the trajectories over time of both the learned association and the behavioral outcomes. Another critical issue entails whether it is necessary and sufficient that volunteers express a desire for change in their behavior for sleep learning to occur. Indeed, all participants in Arzi et al. (2014) study, who demonstrated a reduction in number of smoked cigarettes following sleep learning, had expressed their willingness to quit smoking. Since motivational factors seem to enable sleep-related memory consolidation (Rasch and Born, 2013), the outcome for populations whose volition is either not strongly exercised (e.g., young children, dementia patients) or needs to be challenged (e.g., PTSD patients) cannot be predicted at present. Finally, even if the current and hypothesized research and clinical procedures cannot be compared to Watson fear conditioning of the helpless Little Albert (Watson and Rayner, 1920), ethical considerations of learning in states of unconsciousness should be carefully considered.

In summary, the study by Arzi et al. (2014) demonstrated that relatively durable, positive health outcomes can be achieved via odor-based associative learning during sleep without any conscious effort by the participants. Their results hold far-reaching implications not only for future neuroscientific investigations within the fields of sleep, olfaction, emotion, and memory, but also for the clinical domain. We hope that these promising data will inspire researchers to elaborate on the possibilities of this implicit learning mechanism, bearing in mind the unresolved ethical and methodological issues.

## Conflict of interest statement

The authors declare that the research was conducted in the absence of any commercial or financial relationships that could be construed as a potential conflict of interest.
